# Gender equity in anesthesia: is it time to rock the boat?

**DOI:** 10.1186/s12871-023-01987-4

**Published:** 2023-03-08

**Authors:** M Gisselbaek, OL Barreto Chang, S Saxena

**Affiliations:** 1 Department of Anesthesiology and Acute Medicine, Geneva University Hospitals, Geneva, Switzerland; 2 Department of Anesthesia and Perioperative Care, University of California San Francisco, San Francisco, CA USA; 3 Department of Anesthesia and Reanimation, AZ Sint-Jan Brugge Oostende AV, Brugge, Belgium

**Keywords:** Gender equity, Leadership

While gender inequalities have been reduced over the last decades, the United Nations women’s report recently showed that women are still restricted from working in certain industries in almost 50% of countries (based on a sample from 93 countries) [1]. On top of that, essential women’s rights, like the right to abortion, have been withdrawn from the United States constitution [2, 3], obstructing the much-needed road to gender equity. Gender equity, which we are discussing here, is about giving everybody the tools to succeed regardless of gender [4].

The medical profession has not yet achieved gender equity, especially in high positions. Despite equal representation in medical schools [5], women remain a minority in meaningful academic and leadership positions, which may be explained by unconscious bias, motherhood penalty, and impostor syndrome [6]. In 2019, gender salary gaps and representation were still prevalent among academic medical specialties in the US, with women representing only 24% of full-time professors [7].

A recent transcontinental analysis of gender disparity at the top 25 medical schools highlighted the underrepresentation of women in senior leadership positions, such as department chairs, clinical and non-clinical professors, and assistant professors (based on rank and publication index) [8]. Mentorship has been identified as an important steppingstone. However, fewer women mentors exist past the assistant professor rank, leading to a need for more identification and representation by female anesthesiologists [9]. The lack of representation at the higher levels of academic and professional ranks leads to a scarcity of identification, often discouraging women. For instance, a recent survey of European anesthesiologists showed that women are less determined to obtain leadership positions than their counterparts even though they are equally interested in research [10]. Global societal stereotypes, such as cultural expectations about family-life and the lack of respect for personal time within neo-liberal academics that may disfavor women’s evolution, could explain this phenomenon [8]. Medical culture still celebrates competitive individualism and masculine norms of leadership [11]. Gender hierarchy is also strengthened by gender stereotypes as a women’s leadership style is often feminine and criticized for being “status incongruent” [12], which can be repercussed on their evaluations.

While some progress has been made by documenting gender imbalance, increasing the number of female politicians, improving gender-equality in sports, and eliciting policies to decrease the consequences of gender inequality [13], the recent covid-19 pandemic challenged these efforts.

During the covid-19 crisis, the increased need for childcare put a strain on working parents, with mothers carrying a heavier mental and actual load than fathers [14]. Moreover, the lack of household assistance due to social distancing during the pandemic directly hindered women’s academic productivity [15].

Recently, interest in gender studies has increased with, some of the best anesthesiology journals dedicating special issues to women [16] and societies dedicating committees to achieve gender equity [17]. Panels and workshops at anesthesia conferences included an increasing number of women; this was seen among the major conferences worldwide, such as the 2022 European Society of Anaesthesiology and Intensive Care (ESAIC) Meeting [18] and at the 2022 American Society of Anesthesiologists (ASA) Conference [19].

Unfortunately, some disparities are inherent to the current state of academic anesthesia [20]. A practical example can be found in the editorial board of current anaesthesiology journals, as there are significantly fewer women [21–23]. Somehow, explicit bias, such as the mother penalty, and implicit or unconscious bias such as lack of men-attributed leadership traits, may hinder professional progression. Moreover, personal implicit barriers such as impostor syndrome may add to the inequalities reported [24].

In this editorial, we address different types of gender bias and provide suggestions on how to overcome these. (Fig. 1).

**Fig. 1:**
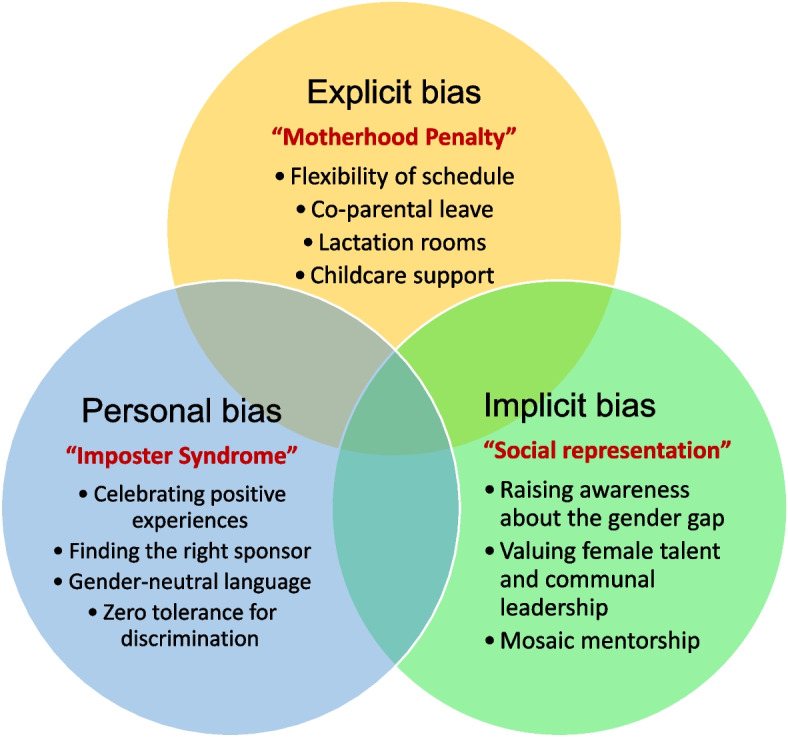
Suggestions to overcome explicit, implicit and personal bias

## Explicit bias: “Mother penalty”

Physicians, particularly anesthesiologists, must go through many years of medical training, which is often not considered as an ideal period for parental leave.

Parental leave, pressure from learning objectives, and numerous hours spent at work often discourage female residents from having children and make them postpone their parental goals. On top of that, parental leave is not shared in most countries. Past residency, women with academic goals often choose to set aside their family wishes to compete with men in the same position, setting aside their personal family goals.

Women in leadership positions are prone to choose a different lifestyle than their counterparts and are therefore less likely to have children [6]. Program directors in anesthesiology tend to think that pregnancy and taking a parental leave negatively affect women’s residencies by impacting timeliness, scholarly activities, technical skills, training experiences of co-residents, and opportunities for fellowship when compared with fathers [25]. A recent survey on residents’ perceptions of parental leave in anesthesiology in the US showed that female residents are more affected than male residents when welcoming a child [26]. Moreover, in the same study, they felt that delaying childbirth until the end of residency raised infertility issues and that breastfeeding often competed with their tasks.

Achieving gender equity goes past improving physician parenting experiences. Gender bias is linked with the expectation that women set aside their professional growth to take parental leave, often missing promotions. These biases not only disadvantage women, but also men who would like to take time off for childcare. To date, only a few countries, such as Scandinavian countries, comply with items making work family-friendly (e.g. flexibility of schedule, co-parental leave, and childcare support systems). One of these items also consists of lactation rooms, as recommended in 2021 by the ASA [27]. A current work culture change should be implemented to create a much-needed support-system for mothers, fathers, and breastfeeding anesthesiologists.

## “Implicit bias: male-attributed leadership skills”

Implicit gender bias describes the unconscious and automatic preconceived idea about one gender, coming from culture, norms, and values. This bias has a significant influence on the way we perceive leaders. Even if studies show that gender doesn’t influence the ability of one to perform in a leadership position, female leaders often lack perceived legitimacy [8, 28]. Indeed, leaders are often expected to present “agentic” qualities (assertive, confident, independent), which are often more masculine behaviors. These contrast with “communal” qualities (cooperative, team-focused, empathic), which are often perceived as more feminine and fit less the archetypical norm for professional success [29]. Women in leadership positions find themselves in incongruent roles. Role congruity theory is a concept where society will positively evaluate a person when one aligns with their expected role [30]. In line with this concept, research finds that women leaders are often evaluated as having either an agentic deficiency (viewed as lacking the competence to be a leader) or an agentic penalty (penalized for their display of dominance) [12]. They find themselves in a double bind/dilemma, juggling between the communal qualities people prefer in women, and the agentic qualities people think leaders need to succeed. For example, ideal code leadership is embodied by highly agentic, stereotypical male behavior. Female gender stereotypes may conflict with such behavior, finding women challenged in finding alternative strategies to integrate this dual identity [31]. Implicit bias goes past leadership roles. The language used (or omitted) to describe an individual is strongly influenced by social norms [29]. This gendered language in a professional context reinforces masculine professional stereotypes and may hinder women’s career aspirations or recognition.

Work satisfaction and success are achieved when work aligns with one’s values. This conflict of social values may explain why women, with same professional aspirations as their male counterparts progressively make choices resulting in disparities in career path, a phenomenon represented by the metaphor of the leaky pipeline. Women tend to orient their careers towards fields such as education or community improvement (more communal) than academic or clinical fields (more competitive). Yet another masculine cultural bias influences the perception of these career choices. According to the “Pollution theory”, when fields better align with traditionally feminine values or become more represented by the female gender, they progressively lose in salary and prestige [32]. Without awareness of this phenomenon, women’s contributions will continue to be undervalued [11].

Raising awareness about unintentional social constructs and stereotypes (often incompatible with one’s values) is essential to learn to identify and prevent gender biases. This will further teach us to acknowledge alternative leadership styles compatible with the emerging idea that healthcare leadership is about team play.

## “Personal bias: Impostor syndrome”

Impostor syndrome is “the inability to internalize success and the tendency to attribute success to external causes such as luck, error or knowing the appropriate individuals” and is known to happen in high-achieving women [24, 33]. Impostor syndrome (IS) issues have recently been raised within medicine. Medical culture, perfectionism, and individual blame might enhance these feelings [34]. Female gender, low self-esteem, and institutional culture are associated with the emergence of IS in physicians and physicians in training, which is associated with higher rates of burnout [24]. Burnout within the medical profession has been documented to occur more often in women than men [35], which leads to increased medical leave periods, decreased performance, and might disadvantage women. Additionally, female anesthesiologists in the operating room report being more mistreated and undermined by surgeons than their male counterparts [10], possibly magnifying IS issues. These personal biases are likely to result from the institutional culture and global societal stereotypes. Impostor syndrome is too often used to justify why gender bias is difficult to overcome [36, 37]. Indeed, it is important to keep in mind that the barrier to women’s growth is frequently systematic [37, 38].

Even though impostor syndrome is an internal feeling, it remains important to help physicians and women find ways to overcome it. Validation of success, institutional support, mentoring strategies, and positive affirmation have been found to decrease IS [24, 39]. Moreover, the simple fact of acknowledging that the feeling exists might be beneficial. Creating a medical culture that gives physicians space to share their struggles is essential [40].

## General recommendations

Recommendations on how to overcome gender bias exist. For instance, the American college of physicians recently released a list of the top 10 things to do to reach gender equity [41]. Raising awareness, zero tolerance policies, promoting parental leave and advocating for family, amplifying accomplishments and celebrating positive experiences of female leaders, helping, sponsoring, and mentoring women are steps to take on the road towards gender equity [6, 41]. Acknowledging and broadcasting the progression of female leaders throughout anaesthesia could encourage and inspire women in their early careers [8]. Moreover, measuring and reporting the progress made toward gender equity is necessary.

## Conclusion

Obtaining gender equity in anesthesiology remains problematic. Promoting different leadership styles and different styles of mentors, by overcoming explicit, implicit, and personal bias, allows the creation of an environment in which everyone attains their full potential. Future research should aim at understanding how societal stereotypes and medical culture create implicit bias that stall the progression of female anesthesiologists. This could lead to the creation of innovative strategies to promote gender equity within medicine.

## Data Availability

Not applicable.

## Abbreviations

ASA: American Society of Anesthesiologists
ESAIC: European Society of Anaesthesiology and Intensive Care
IS: Impostor Syndrome
